# The trans-generational impact of population density signals on host-parasite interactions

**DOI:** 10.1186/s12862-016-0828-4

**Published:** 2016-11-25

**Authors:** Jessica Michel, Dieter Ebert, Matthew D. Hall

**Affiliations:** 1 University of Basel, Zoological Institute, Vesalgasse 1, 4051 Basel, Switzerland; 2 School of Biological Sciences, Monash University, Melbourne, VIC 3800 Australia

**Keywords:** Condition dependence, Crowding-stress, *Daphnia*, Density-dependent prophylaxis, Infectious disease, Life-history, Maternal effects, *Pasteuria ramosa*

## Abstract

**Background:**

The density of a host population is a key parameter underlying disease transmission, but it also has implications for the expression of disease through its effect on host physiology. In response to higher densities, individuals are predicted to either increase their immune investment in response to the elevated risk of parasitism, or conversely to decrease their immune capacity as a consequence of the stress of a crowded environment. However, an individual’s health is shaped by many different factors, including their genetic background, current environmental conditions, and maternal effects. Indeed, population density is often sensed through the presence of info-chemicals in the environment, which may influence a host’s interaction with parasites, and also those of its offspring. All of which may alter the expression of disease, and potentially uncouple the presumed link between changes in host density and disease outcomes.

**Results:**

In this study, we used the water flea *Daphnia magna* and its obligate bacterial parasite *Pasteuria ramosa,* to investigate how signals of high host density impact on host-parasite interactions over two consecutive generations. We found that the chemical signals from crowded treatments induced phenotypic changes in both the parental and offspring generations. In the absence of a pathogen, life-history changes were genotype-specific, but consistent across generations, even when the signal of density was removed. In contrast, the influence of density on infected animals depended on the trait and generation of exposure. When directly exposed to signals of high-density, host genotypes responded differently in how they minimised the severity of disease. Yet, in the subsequent generation, the influence of density was rarely genotype-specific and instead related to ability of the host to minimise the onset of infection.

**Conclusion:**

Our findings reveal that population level correlations between host density and infection capture only part of the complex relationship between crowding and the severity of disease. We suggest that besides its role in horizontal transmission, signals of density can influence parasite epidemiology by modifying mechanisms of resistance across multiple generations, and elevating variability via genotype-by-environment interactions. Our results help resolve why some studies are able to find a positive correlation between high density and resistance, while others uncover a negative correlation, or even no direct relationship at all.

**Electronic supplementary material:**

The online version of this article (doi:10.1186/s12862-016-0828-4) contains supplementary material, which is available to authorized users.

## Background

Environmental forces shaping host-pathogen interactions can potentially influence the outcome of infection in current and subsequent host generations. A given host may experience fluctuations in a range of environmental conditions, including food availability and quality, temperature, habitat quality, and predation [1, 2]. Anticipating this variability, however, an individual is able to change not only his or her own investment in immune defence strategies [3, 4], but also bias the investment strategies of their offspring [5, 6]. Indeed, both paternal and maternal effects have been shown to have a profound impact on the expression of disease [7–10]. Such trans-generational effects not only allow parents to better prepare their offspring for upcoming environmental challenges (but see [11, 12]), but also modify the rate and trajectory of evolutionary change [13, 14].

One common form of environmental heterogeneity is the changes in density that can occur as populations shrink or expand due to reproduction, migration, and mortality. For epidemiological models of disease, these changes in density are a key predictor for the likelihood of transmission. At higher densities, animals are expected to experience elevated levels of parasitism and more frequent epidemics [15, 16], with species living in dense aggregations, such as social animals and high-density livestock, being notorious for the rapid spread of disease [17, 18]. Changes in density, however, not only increase the likelihood of transmission, but also influence a host’s immune system. Under the *density-dependent prophylaxis hypothesis*, high density is predicted to favour hosts that increase resource allocation in immune defence, thereby enhancing resistance in the face of elevated parasitism [19]. Yet, high density often coincides with stressful conditions, such as low food levels, increased exposure to metabolic waste, and physical interference. Consequently, the *crowding stress hypothesis*, predicts that hosts living in high-density conditions are more stressed and thus more vulnerable to infection due to a down regulation of the immune system [20, 21].

Taken in isolation, the density-dependent prophylaxis and crowding stress hypotheses make contrasting predictions with regard to an individual’s ability to defend against infection under crowded conditions. Hosts are predicted to become more resistant if investment in immune defences is increased [19, 22], or less resistant if the stress of a crowded environment causes the condition of the host to decline [20, 21]. A simple change in the average resistance of a population, however, belies the complexity of the density and resistance relationship. Due to the anticipatory behaviour of parents, the influence of density changes can extend beyond the immediate generation [23], particularly if signals in the parental conditions are indicative of the offspring environment [11]. The strategy that an individual host adopts may also depend on their own overall quality or condition (sensu [24]), leading inevitably to variation across individuals, genotypes, and populations, in whether average susceptibility increase or decreases.

Here we test how host and pathogen genotypes of different quality respond to experimental conditions that signal low and high density, and if parents and their offspring cope with infections in the same manner. Studies have increasingly recognised how different genotypes can vary in their response to environmental conditions [25], and it is highly likely that changes in density signals, like many other environmental characteristics, will be involved in specific genotype-by-environment interactions (G x E). More difficult to predict is how the density-dependent prophylaxis hypothesis or crowding stress hypothesis is modified by the previous generations experiences. Under the density-dependent prophylaxis hypothesis we might expect that high density signals experienced by parents result in more resistant offspring, while the opposite would be true if the crowding stress hypothesis applies. However, the crowding stress hypothesis seems at odds with the common observation that poor maternal environments often promote increased offspring resistance [7, 26, 27]. Our goal was to extend the study of host density and infection outcomes to include the common evolutionary concepts of trans-generational effects and genotype-specific responses.

The study system for this experiment was the freshwater crustacean *Daphnia magna* and its bacterial pathogen, *Pasteuria ramosa. Daphnia* are well known for their plastic life-history and morphological responses to environmental parameters like predation [28, 29], pollutants [30], temperature [31, 32], salinity [33], and food stress [34, 35]. As an inhabitant of rock-pools and ponds, they also experience extensive and fast variation in population density across the season, with numbers ranging from a few females per cubic meter to up to 1000 *Daphnia* per litre [36, 37]. In response to signals of high density, *Daphnia* have been shown to reduce filter-feeding rates, growth and offspring number [38–40]; instead producing offspring of a larger size or switching to sexual reproduction in some circumstances [41]. Whether or not this translates to disease resistance remains unclear, but when combined with food stress, a crowded environment can potentially reduce infection rates (but see [26, 42]).

One of the challenges of studying population density is disentangling the various signals that are responsible for a host’s awareness of density. Increases in physical interference and metabolic waste, and a reduction in food availability, are all consequences of crowding. Here, we were able to isolate only the chemical signals of crowding by raising animals individually in jars using “conditioned water” from crowded *Daphnia* cultures (see [39]). By filtering the water first, and then reintroducing food in a controlled manner, we thus avoided the confounding influences of food stress, filter-feeding rates, and physical interference. Using this conditioned water, we conducted two cross-infection experiments (two host and two parasite genotypes), to study the direct (F_0_ generation) and maternal (F_1_ generation) effects of crowding on infection rates, host fecundity and body size (fitness traits for the host) and parasite spore load (fitness traits for the parasite). Our aims were: i) to test for the effect of a chemical signal of crowding on disease trait expression, without the crowding-associated effect of food stress; ii) to test for genetic variation in disease expression across four combinations of host and parasite genotypes; and, iii) to compare patterns of host resistance to infection in both the parental and offspring generations.

## Methods

### The study system

*Daphnia magna* Straus is a freshwater crustacean found in standing freshwater ponds and lakes throughout Eurasia and North America. *Daphnia* reproduce via cyclical parthenogenesis and feed on small substrates in the water, commonly planktonic green algae. They are hosts for a variety of parasites [43], including the endospore forming bacteria, *Pasteuria ramosa* Metchnikoff 1888. When an infection takes place, the parasite castrates the host within five to 15 days, the host’s body size increases (i.e. parasite induced gigantism), and up to 20 million spores accumulate in the body cavity [44]. Transmission is exclusively horizontal with spores released from the decaying cadaver of infected animals [44].

The two host and parasite genotypes used in this study were chosen as they are completely compatible and differ strongly in characteristics of the onset and severity of infectious disease [35, 45, 46], enabling a mechanistic test for the influence of density manipulations on host-parasite genotypes across generations. The host genotype HO2 originates from Hungary, and genotype M10 originates from Belgium, while the two parasite genotypes were C1 originating from Moscow, Russia; and C19, derived from North Germany. Prior to the experiment, all *Daphnia* clones were kept under standardized conditions for three generations. They were raised individually in 100-mL jars filled with 80 mL of artificial media (ADaM, [47]) and kept in a single controlled climate chamber (16:8 light–dark cycle and 20 °C). Animals were fed daily with algae (*Scenedesmus sp.*) and food levels were gradually increased to meet the growing needs of the animals, from 0.5 million cells per animal per day at birth to 8 million cells per animal per day from age 13 days onwards.

### Manipulation of host density signals

In a variety of zooplankton, including *Daphnia*, previous studies have established that info-chemicals released by individuals, presumably metabolic waste, can influence the life-history investment of others in the population [38–40, 48]. This has been discovered by culturing *Daphnia* at different densities and then exposing new individuals to the same “conditioned” media (after filtering or removing the *Daphnia* and their leftover food). Here we use this approach to simulate: i) a high-density population that is typical of crowded conditions; and, ii) an non-crowded or low-density population, that is representative of a pond in the early season and similar to the typical conditions of most *Daphnia* lab studies (thereby a useful reference point).

Each treatment consisted of 12 × 1.5-L jars that were provided with 1000 million cells of algae per day and maintained without aeration. For the high-density treatment, every jar contained 250–300 adult *Daphnia* L^−1^ of mixed age and genotype (HO2 and M10 mixed). For the low-density control group, no animals were present but the jars were maintained the same otherwise. Water from these jars was collected every three days by using a coarse-meshed plankton net (mesh size 0.1 mm) to remove any *Daphnia*, and then pumping the conditioned water through a 0.45 μm filter to remove debris and algae cells. The jars were filled again with fresh ADaM media, and the number of animals in the high-density treatments adjusted once again to 250–300 adult animals L^−1^.

### Cross-infection experiments

Cross-infection experiments were conducted across two generations in order to study how signals of density influences infection outcomes in mothers, as well as their offspring. In the parental generation (F_0_), we explored the direct effect of crowding signals by raising animals individually in the high- or low-density conditioned water from birth. We used a full-factorial design where in each of the two different conditioned waters, we exposed two host clones (HO2 and M10) to two parasite clones (C1 and C19). In a second experiment, we then studied the maternal effects of this crowding manipulation on host-pathogen interactions. Mirroring the same factorial design as in the parental generation, offspring of both host genotypes (HO2 and M10) were collected from the low-density and high-density control mothers (i.e. unexposed to any pathogen), and then exposed to the two parasite clones (C1 and C19). All animals in the offspring generation (F_1_) were raised under standard artificial media, leaving the maternal effect of high- and low-density conditioned water as the only environmental manipulation.

In each generation (F_0_ and F_1_), the cross-infection experiment consisted of eight treatment groups (2 host genotypes x 2 parasite genotypes x 2 direct or maternal environments) with 42 replicates for each group (336 animals) and a control-group without parasites for each condition with 28 replicates each (2 host genotypes x 2 water qualities, 112 animals) leading to a total of 448 animals. To begin an experiment, animals were collected daily from their standardized cultures (parental generation), or manipulated mothers (offspring generation) and maintained in a mass culture. On day three, they were randomly allocated to the treatments and placed individually in 100-mL jars, filled with 20 mL of the appropriate treatment media. On days 4 and 5, animals received either 10 000 spores of the appropriate exposure group (parasite genotypes C1 or C19) or the equivalent volume of a control (placebo suspension produced from uninfected *Daphnia*). The spore dose was chosen based on previous work with the same genotype combinations, which demonstrated infection rates of between 40 and 80% across a range of benign or stressful environmental conditions [27, 33, 35]. On day 6 the *Daphnia* where transferred to fresh jars containing 70 mL of the appropriate media. The animals were fed daily with algae, keeping food levels equal amongst all treatments (as above), and transferred to new media every three days. All jars were maintained in the same incubator and their position changed daily to minimise any positional effects.

We monitored animals daily for survival, with the dead recorded and frozen at −20 °C for further investigation. We recorded the size of every clutch until the end of the experiment (28 days post-exposure). Then host body size was measured using a scaled binocular microscope and each *Daphnia* was frozen individually in 500 μL ADaM for subsequent inspection of infection status and parasite spore quantification. We determined spore loads using an Accuri C6 flow cytometer (BD Biosciences, San Jose, USA). In one counting round, 24 *Daphnia* samples were defrosted, crushed with a pestle and then vigorously mixed with a vortex shaker. Half a PPE 96 well plate was loaded with 190 μL of 5 mM EDTA and then mixed with 10 μL of the crushed-*Daphnia* ADaM solution. Each spore population was counted twice using a custom gate based on fluorescence (FL3) and side scatter (SSA) channels and the average of these values was used in the subsequent analyses.

### Data analysis

All statistical analyses were performed in R (ver. 3.0.1; R Development Core Team, available at: www.r-project.org). Traits were analysed using a full-factorial analysis of variance (Type III) with either two main effects (control data: density and host clone) or three main effects (infection data: density, host clone and pathogen clone). Infection rate (proportion of animals infected) was analysed using a generalized linear model with a logit link function. The models for host fecundity (total offspring per female, square-root transformed), host size (body size at 28 days post-infection), and parasite spore load (parasite spores per female) were fitted using least squares. To avoid confounding estimates of host and parasite performance with survival, only individuals that survived until the end of the experiment were used in the analysis of offspring production, spore counts, and body size. Due to differences in average survival and infection rates, as well as handling errors, sample sizes for genotype HO2 varied between 21 to 38, and for M10 between 26 and 37, but there was no confounding effects of the direct or maternal density manipulations on the samples sizes (see Additional file 1). Finally, relative effect sizes were calculated as partial eta-squared values, which estimate the proportion of variability associated with a particular effect, after controlling for the variance associated with all other effects (e.g. partial-η^2^ = SS_effect_/[SS_effect_ + SS_error_]).

## Results

### The response of *Daphnia* to the manipulated signals of crowding

**Fig. 1:**
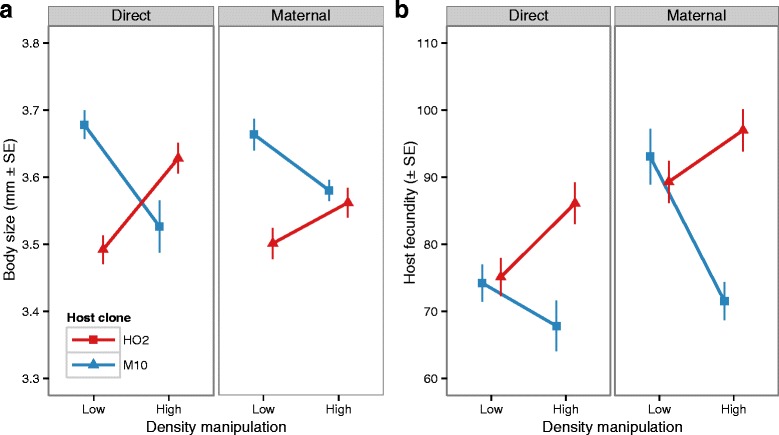
The influence of high and low signals of population density on the (**a**) body size and (**b**) fecundity of unexposed, control *Daphnia*. For each trait the left graph shows the results from the directly exposed individuals (F_0_ generation), while the right graph shows the results for the maternally manipulated offspring (F_1_ generation)

We first examined the impact of low-density and high-density conditioned water on host body size and fecundity across two generations in the absence of parasitism. Overall, the observed patterns were consistent between the direct (F_0_) and maternal (F_1_) manipulations of population density, but the impact of conditioned water on body size and fecundity was strongly host clone specific (Table 1: all G_H _x E, *p* < 0.05). As a direct response to high-density conditioned water, host clone M10 increased in body size and fecundity, while the reverse pattern was observed for clone HO2 (Fig. 1a). The nature of this host clone specific response was maintained in the subsequent generation. In response to the maternal manipulation, the offspring of mothers from the high-density treatments altered their body size and fecundity in the same direction as their mothers (Fig. 1b), even when raised in the control, low-density conditioned water.

**Table 1:** Results of the analyses describing the effects of host genotype, and density manipulation on the life-history characteristics of unexposed, control animals

	Direct effect (F_0_)		Maternal effect (F_1_)	
Body size	*F*_1, 94_	Partial-η^2^	*F*_1, 85_	Partial-η^2^
Host clone (G_H_)	2.40	0.025	17.79***	0.173
Density effect (E)	0.08	0.001	0.28	0.003
G_H_ x E	27.82***	0.228	11.30***	0.117
Overall fecundity	*F*_1, 94_	Partial-η^2^	*F*_1, 86_	Partial-η^2^
Host clone (G_H_)	9.13**	0.088	11.25**	0.116
Density effect (E)	0.22	0.002	4.35*	0.048
G_H_ x E	7.62**	0.075	18.13***	0.174

Direct effect refers to the treatment where animals were raised directly in conditioned water, whereas the maternal effect refers to the offspring of these manipulated mothers. Traits measured include the body size and lifetime fecundity of animals that survived until the end of the experimental period. Presented are the appropriate test statistics (*p < 0.05, **p < 0.01, ***P < 0.001), and relative effect sizes (partial-η2) that estimate the proportion of variability associated with a particular effect

### The direct effects of density signals on host-parasite interactions

**Fig. 2:**
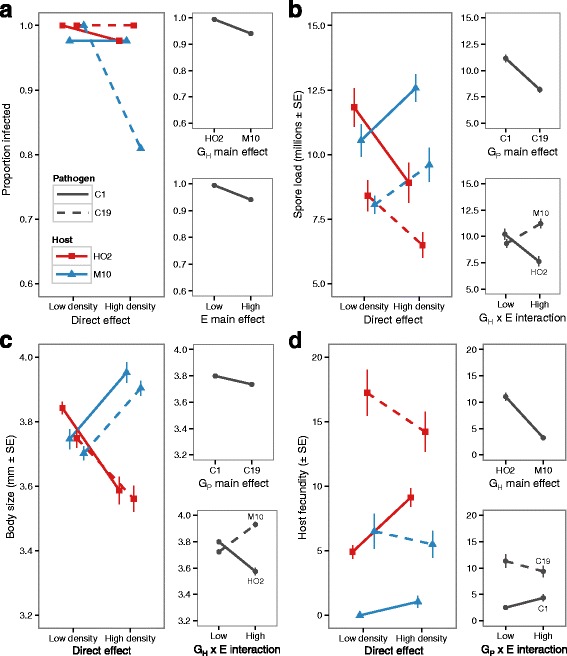
The direct effect of low- and high-density manipulations on (**a**) infection rates, and the (**b**) spore loads, (**c**) body size and (**d**) host fecundity of infected animals. All animals were raised directly in the conditioned water. For each trait the tall graph shows all eight treatment combination, while the smaller graphs to the right show the treatment effects with the largest effect size (see Table 2)

We found that raising animals in either low-density or high-density conditioned water had little impact on infection rates (Fig. 2a). Except for one treatment group (high-density, pathogen C19 and host M10, infection rate = 0.81), almost all exposed animals became infected (average infection rate = 0.99). This lack of variability prevented a quantitative evaluation of the infection data (as per Table 2), but there appears to be no clear relationship between host-density manipulations and infection rates. For all other traits, however, the impact of the density manipulation depended on both host genotypes (G_H_), parasite genotypes (G_P_) and their interactions with the density environment (G_H_ x E or G_P_ x E). Notably there was no independent effect of host-density signals directly on disease characteristics, and very little variation in disease attributed to this factor alone (i.e. low partial-η2 values, Table 2).

**Table 2:** Results of the analyses describing the effects of host genotype, and density manipulation on the disease characteristics of infected animals

	Direct effect (F_0_)		Maternal effect (F_1_)	
Infection rate	*χ* ^2^_1_	Partial-η^2^	*χ* ^2^_1_	Partial-η^2^
Pathogen clone (G_P_)	–	–	2.08	0.013
Host clone (G_H_)	–	–	7.25**	0.042
Density effect (E)	–	–	8.22**	0.048
G_P_ x G_H_	–	–	0.40	0.002
G_P_ x E	–	–	2.40	0.015
G_H_ x E	–	–	0.27	0.002
G_P_ x G_H_ x E	–	–	0.31	0.002
Pathogen spore loads	*F*_1, 220_	Partial-η^2^	*F*_1, 224_	Partial-η^2^
Pathogen clone (G_P_)	42.28***	0.161	3.77^#^	0.017
Host clone (G_H_)	8.75**	0.038	4.89*	0.021
Density effect (E)	0.53	0.002	4.15*	0.018
G_P_ x G_H_	0.05	<0.001	6.16*	0.027
G_P_ x E	0.08	<0.001	0.01	<0.001
G_H_ x E	23.41***	0.096	0.12	0.001
G_P_ x G_H_ x E	0.73	0.003	3.19^#^	0.014
Host body size	*F*_1, 241_	Partial-η^2^	*F*_1, 229_	Partial-η^2^
Pathogen clone (G_P_)	6.36*	0.026	7.52**	0.032
Host clone (G_H_)	45.45***	0.159	1.25	0.005
Density effect (E)	0.17	0.001	25.78***	0.101
G_P_ x G_H_	0.11	<0.001	4.53*	0.019
G_P_ x E	0.57	0.002	2.60	0.011
G_H_ x E	103.02***	0.299	22.84***	0.091
G_P_ x G_H_ x E	0.76	0.003	0.60	0.003
Overall host fecundity	*F*_1, 241_	Partial-η^2^	*F*_1, 230_	Partial-η^2^
Pathogen clone (G_P_)	89.26***	0.270	14.84***	0.061
Host clone (G_H_)	200.18***	0.454	35.82***	0.135
Density effect (E)	2.47	0.010	17.00***	0.069
G_P_ x G_H_	1.05	0.004	0.73	0.003
G_P_ x E	6.18*	0.025	3.08	0.013
G_H_ x E	0.13	0.001	0.34	0.001
G_P_ x G_H_ x E	1.41	0.006	2.02	0.009

Direct effect refers to the treatment where animals were raised directly in conditioned water, whereas the maternal effect refers to the offspring of these manipulated mothers. Traits measured include the proportion of animals infected overall, plus the pathogen spore loads, body size, and lifetime fecundity of animals that survived until the end of the experimental period. Presented are the appropriate test statistics (^#^ *p* < 0.1, *p < 0.05, **p < 0.01, ***P < 0.001), and relative effect sizes (partial-η^2^) that estimate the proportion of variability associated with a particular effect

Both pathogen spore loads and host body-size were influenced by pathogen genotype and an interaction between host genotype and density (Table 2: G_P_ and G_H_ x E). While the fecundity of infected animals depended on the host genotype and an interaction between pathogen genotype and density treatment (Table 2: G_H_ and G_P_ x E). The complexity of these patterns are shown in Fig. 2. Here, quality differences between the host and pathogen genotypes are observed, with pathogen C1 consistently producing the most spores (Fig. 2b), and host HO2 the most offspring (Fig. 2d). Yet the most interesting patterns occur when shifting from the low-density to the high-density treatment. Only in the high-density treatment were differences between the *Daphnia* clones observed. The spore loads and body sizes of infected M10 genotypes increased in this environment, whereas the reverse pattern occurred for host genotype HO2. Independently, pathogen C1 was the more efficient castrator of infected hosts (lowest fecundity), but the differences between the pathogen genotypes decreased in the high-density environment.

### The maternal effects of density signals on host-parasite interactions

In the subsequent generation, we found that the environment experienced by the mothers significantly affected the traits of their offspring. Unlike the direct influence of density on host and parasite traits, the maternal influence of crowding was largely independent of the effect of host and pathogen genotypes, and accounted for much more of the variability in disease (higher partial-η2 values, Table 2). Infection rates varied with both the genotype of the host and the density environment of the mother (Table 2: G_H_ and E). The independent effect of the maternal environment was also observed for pathogen spore loads (Table 2: together with and interaction between host and pathogen genotypes, E and G_P_ x G_H_) and host fecundity (Table 2: together with host and pathogen genotypes, E, G_P_ and G_H_).

**Fig. 3:**
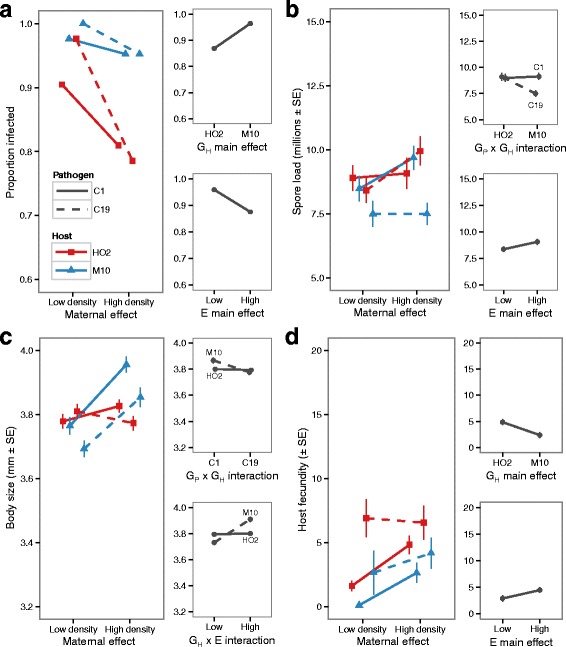
The maternal effect of low- and high-density manipulations on (**a**) infection rates, and the (**b**) spore loads, (**c**) body size and (**d**) host fecundity of infected animals. All animals were the offspring of mothers raised in the conditioned water. For each trait the tall graph shows all eight treatment combination, while the smaller graphs to the right show the treatment effects with the largest effect size (see Table 2)

As shown in Fig. 3, animals whose mothers experience the signals of a high-density environment had lower rates of infection (Fig. 3a: 10% lower success), and when infected produced marginally more offspring (Fig. 3d, 1.6 times more offspring on average) and pathogen spores (Fig. 3b, almost 1 million more spores). For host body-size, however, the impact of the maternal density effect varied with host genotype, in combination with an interaction between host and pathogen genotypes (Table 2: G_H_ x E and G_H_ x G_P_). Only host genotype M10 appeared to be influenced by density and pathogen genotype (Fig. 3b), with the largest body-size occurring for those animals infected by C1 in the high-density maternal environment treatment.

### The multivariate responses of infected hosts under crowding

**Fig. 4:**
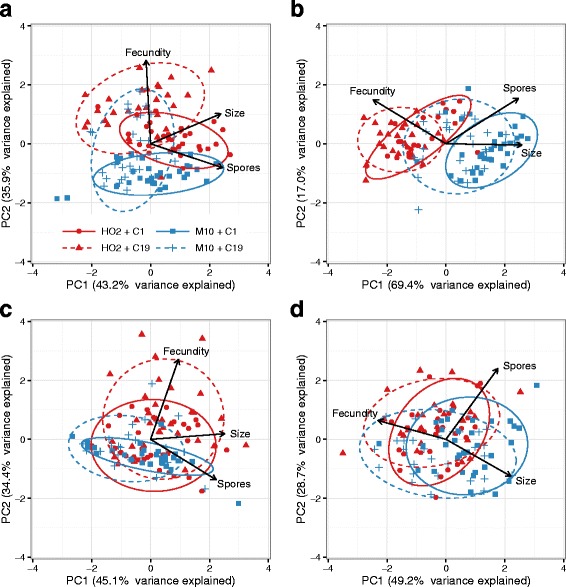
The multivariate responses of infected hosts to low- and high-density population signals. Each panel represents a different principal component analysis conducted using individuals with complete data for fecundity, body size, and spore loads. Before analysis traits were standardised to a mean of zero and standard deviation of one. The loadings of each trait to the relevant multivariate axes are indicated by the direction and angle of each traits vector (the arrows). Ellipsoids indicate treatment group clustering based on a multivariate t-distribution and 95% confidence level. **a** Direct effect & Low − density. **b** Direct effect & High − density. **c** Maternal effect & Low − density. **d** Maternal effect & High − density

To formalise how the relationships amongst symptoms of disease under signals of low- and high-density, we conducted a principal components analysis on each of the four broad treatment combination (direct and maternal effects by low-density and high-density). From these multivariate analyses, two patterns emerge. First, once controlling for variation in trait means (all traits within each subset were standardised first), the broad relationships amongst traits within the low-and high-density treatments were very similar (Fig. 4a, and c versus b and d). Low-density treatments seem to be defined by variation in fecundity (PC2) and independently positive correlations between body size and spore loads (PC1); whereas, the high-density manipulation is characterised by a trade-off between fecundity versus spore loads and body size (PC1). Second, the differentiation amongst the host-pathogens combinations is greater when directly exposed to signals of population density (Fig. 4,a and b versus c and d). Mirroring the results of Fig. 1, when directly exposed to low-density signals, different host-pathogen combination are largely separated by variation in host fecundity (Fig. 4a, PC1). High-density exposure sees a shift in each host genotypes response, with host HO2 associated with higher fecundity, and lower spore loads and body size than genotype M10 (Fig. 4b, PC1). In contrast, there is more overlap in the offspring generation, indicating that the maternal effect of density is not necessarily resulting in correlated life-history shifts (Fig. 4c and d).

## Discussion

High population density can have a profound impact on the outcome of disease [15]. With increasing density, individuals are predicted to experience an elevated risk of disease transmission, in combination with increased competition, lower food abundance, and a decline in environmental quality. In response, the average resistance of a population is predicted to either increase as a counter to the elevated threat of parasitism [19, 22], or decrease if the increased stress of a crowded environment reduces the general condition or vigour of a host [20, 21]. At the level of the individual, however, health and performance is shaped by many different factors – from the conditions experienced by their parents, through to their own innate genetic quality and current environmental conditions. Here, we consider how trans-generational effects, and the individual responses of host or pathogen genotypes, can reveal new insight into the functional relationship between host density and infection outcomes.

### Do the signals of population density experienced by a previous generation matter?

Anticipating changes in environmental conditions allows an individual to adjust not only their own life-history and immune investment strategies, but also that of their offspring. Our results reveal that changes in population density induce phenotypic changes that are maintained in the next generation even after the density signal is removed. Using animals from the unexposed treatment (i.e. the controls), we found that each host genotype responded differently to the direct density manipulation (G_H_ x E). In the absence of a pathogen, for example, host clone M10 increased offspring production and growth (i.e. body size at age 30 days) when directly exposed to the chemical signal of crowding, while clone HO2 displayed the reverse pattern (Fig. 1a). In the subsequent generation, the offspring from M10 mothers raised in the high-density treatment were again larger and more fecund than the HO2 offspring (Fig. 1b).

Of particular interest is how the direct response of a host to high-density signals (F_0_), as well as the response of their offspring (F_1_), was consistent in direction and magnitude. The high-density environment increased the relative differences amongst genotypes in both generations, with one clone consistently outperforming the other under signals of crowded conditions, but not under low-density conditions. If these results hold true when expanded to more genotypes, then shifts from crowded to non-crowded conditions can potentially increase or decrease the evolutionary potential of traits across multiple generations (sensu [49]). Whether or not these changes are due to maternal investment or epigenetic modification cannot be determined from our two-generation experimental design. Yet perceiving the chemical signals of crowding alone, as opposed to the associated physical interference or food stress, appears to consistently shape genotype-specific responses within and across generations.

### Can the relationship between population density and host resistance be genotype-specific?

Increases in host density are commonly associated with a change in a population’s resistance to infection. By manipulating the density that an individual experiences, and then characterising aspects of host fitness such as mortality, fecundity or body weight, studies have shown that many populations on average, perform better when infected after being raised in crowded conditions [22, 50–53]; but a negative correlation, or even no direct relationship, between the immediate effects of density and the severity of infection is also possible [21, 54, 55]. Indeed, our results indicate that an appropriate response to signals of density, like many other environmental variables such as temperature or food quality, can depend on the genotype of the host or parasite (see [25, 31, 56]). Upon directly experiencing the signals of high density, for example, host genotype HO2 followed the expectations of the density-dependent prophylaxis hypothesis, reducing the ability of the parasite to proliferate under high-density conditions (lower spore loads, higher fecundity and reduced gigantism, Fig. 4b). Conversely, genotype M10 experienced more severe castration by the parasite (higher gigantism) and greater parasite spore loads (Fig. 4b), akin to the reduction in host resistance predicted by the crowding stress hypothesis. Pathogen genotypes also behaved differently when directly exposed to high-density conditions, with virulence (the reduction in host fecundity) increasing for C19 and decreasing for C1 (Fig. 2d).

Although our use of novel host-pathogen combinations may have exaggerated the observed responses relative to naturally occurring combinations, the presence of either G_H_ x E or G_P_ x E still serves to highlight how density-disease correlations at the population level can overlook the diversity of strategies that different host or pathogen genotypes are adopting. If broadly applicable, these genotypic-specific shifts could permeate through many aspects of host-pathogen coevolution. With higher population densities comes the increased risk of parasitism as well as faster and more frequent epidemics [15, 16]; all things which should accelerate host-pathogen coevolutionary outcomes. Our results add another layer of complexity to the influence of population density, in that signals of crowding may change the relative differences amongst genotypes, dampening or accelerating evolutionary change depending on how genetic variation in host and pathogens is constrained or exposed. We suggest that changes in population density, particularly those mediated by chemical signals, can still have a functional role in shaping the outcome of infection, even if the overall mean resistance of a population remains unaffected.

### How similar are the direct and maternally-mediated changes in disease characteristics?

Maternal effects are known to strongly impact on the ability of a host to fight infection for a range of vertebrate and invertebrate species [7, 8, 57, 58]. Indeed in *Daphnia*, Mitchell and Read [26] showed previously that mothers from a poor environment of high density and low food, produced offspring that were less susceptible to infection. Yet, Ben-Ami et al. [27] suggested that low food alone might account for this effect [see also 35]. Our results show that chemical signals of high density alone, without the confounding effects of resource limitation and physical encounter rates, are sufficient to influence the resistance of individuals in the next generation. However, in contrast to the direct response to high density, which was shaped by genotype-by-environment interactions, the maternal effects were largely unaffected by host and parasite genotypes for infection rates, spore loads, and host fecundity (Table 1 and 2). Moreover, focussing only the effect size of each main factor (G_P_, G_H_ and E), the shift to the offspring generation sees a general reduction in the relative variance explained by host and pathogen genotypes, and an increase in the influence of the crowded environment (partial-η^2^, Table 2).

This contrast between the direct and maternal influence of density suggests that the form of resistance depends on the immediacy of exposure to signals of crowding. A host can fight infection by either minimising the probability of becoming infected, or the severity of disease when a pathogen has established [59]. Here, direct exposure to signals of crowding resulted in shifts in resistance that were driven by changes in the severity of disease alone, via traits such as castration, gigantism and spore loads. Only one out of the four tested combinations of host and genotypes experienced a reduction in infection rates (Fig. 2a), consistent with other studies which have shown that density does not impact directly on the infection success of *Pasteuria* [42, 60]. Instead, one genotype minimised the severity of disease as per the density-dependent prophylaxis, while the other suffered greater fitness loss in concordance with the crowding stress hypothesis. Conversely, offspring born from mothers living in high-density conditions were able to minimise the propensity of the pathogen to infect the host (30% lower infection rate, Fig. 3a), with smaller changes in the severity of castration and parasite proliferation (higher spore loads, Fig. 3b). Here, support for either density-dependent prophylaxis or the crowding stress hypothesis were trait-specific, with infection and fecundity changes supporting the former, and spore load increases the later.

Underlying the direct and maternal responses to crowding, therefore, appears to be either different physiological or immune responses. Hypotheses for the relationship between host density and disease resistance often revolve around the availability of energy available to invest in immune function versus other traits [21, 50]. Yet here, only direct exposure to high-density conditions led to a negative relationship between the fitness of unexposed animals and the resulting consequence of disease once infected. Consistent with the idea that growth in a crowded environment trades off with resistance to infection [3], the host genotype that increased growth and fecundity when uninfected (i.e. M10), suffered most from parasitic castration (lower fecundity, Figs. 2d and 4b) and allowed the parasite to produce more spores (Figs. 2b and 4b). In the subsequent generation, however, the same life-history shifts were maintained in the control animals (higher growth for M10), but now control fitness was uncorrelated with the pattern of host resistance. Thus similar life-history investment does not always predict the same level of resistance, and consequently the mechanistic basic of resistance may be different in the parent and offspring generations.

## Conclusions

While population density has long been recognised as an important driver of infection disease transmission, the influence of crowding has rarely been explored to the same extent as other environmental variables, such as food availability or temperature. Our findings show that density driven changes in host-pathogen interactions will depend on the genotypes involved, how the signal of density are perceived (chemical or physical), and the immediacy of exposure. The outcome of which can be complex, with genotype-specific responses potentially persisting across multiple generations, even when the signal of density is removed. Although our focus here was on symptoms of infection under different signals of density, the true role of the immune system in this response remains to be seen. Immunity is often invoked in both the density-dependent prophylaxis hypothesis and crowding stress hypothesis without assessing any changes in immune gene function. Unravelling the physiological or immune pathways which respond to the chemical signals of crowding will shed light on how changes in population density actually translate into disease outcomes.

## Additional file

Additional file 1: Additional information on samples sizes. Description of data: The breakdown in sample sizes for the analysis of the parental and offspring generations as split by the density manipulation, host genotype, and pathogen genotype. (DOCX 86 kb)
