# Supplementary material for: The trans-generational impact of population density signals on host-parasite interactions
Source: BMC Evol Biol. 2016 Nov 25;16:254. doi: 10.1186/s12862-016-0828-4 (PMC5123254; doi:10.1186/s12862-016-0828-4)
Supplement: Additional file 1: — Additional information on samples sizes. Description of data: The breakdown in sample sizes for the analysis of the parental and offspring generations as split by the density manipulation, host genotype, and pathogen genotype. (DOCX 86 kb) [file 12862_2016_828_MOESM1_ESM.docx]

Additional information on samples sizes

The trans-generational impact of population density signals on host-parasite interactions

Jessica Michel^1,3^, Dieter Ebert^1,4^ and Matthew D. Hall^2,5^

^1^ University of Basel, Zoological Institute, Vesalgasse 1, 4051 Basel, Switzerland

^2^ School of Biological Sciences, Monash University, Melbourne, Victoria 3800, Australia

^3^ j.michel@stud.unibas.ch

^4^ dieter.ebert@unibas.ch

^5^ Corresponding author: [matthew.hall@monash.edu](mailto:matthew.hall@monash.edu)

**Table S1:** Results of the analyses describing the effects of host genotype, pathogen genotype and density manipulation on the survival of animals until the end of the study. Direct effect refers to the treatment where animals were raised directly in conditioned water, whereas the maternal effect refers to the offspring of these manipulated mothers. Presented are the appropriate test statistics for a generalised linear model with binomial data (* p < 0.05, ** p < 0.01, *** P < 0.001).

|  | **Direct effect** | | **Maternal effect** | |
| --- | --- | --- | --- | --- |
|  | ***χ*^2^_1_** | **P-value** | ***χ*^2^_1_** | **P-value** |
| Pathogen clone (G_P_) | 0.005 | 0.947 | 3.470 | 0.062 |
| Daphnia clone (G_H_) | 13.758 | 0.002** | 6.100 | 0.0135* |
| Density effect (E) | 2.091 | 0.147 | 0.487 | 0.485 |
| G_P_ x G_H_ | 1.505 | 0.220 | 0.006 | 0.939 |
| G_P_ x E | 0.160 | 0.689 | 0.044 | 0.833 |
| G_H_ x E | 3.234 | 0.072 | 0.901 | 0.342 |
| G_P_ x G_H_ x E | 0.475 | 0.490 | 0.716 | 0.397 |

**Table S2:** The breakdown in sample sizes for the analysis of the parental and offspring generations as split by the density manipulation, host genotype and pathogen genotype.

| **Density** | **Host** | **Pathogen** | **Exposed (n)** | **Infected (n)** | **Died early (n)** | **Host traits (n)** | **Spore loads (n)** |
| --- | --- | --- | --- | --- | --- | --- | --- |
| **Direct effect of density signal manipulations** | | | | | | | |
| Low-density: | HO2 | *C1* | 42 | 42 | 7 | 35 | 32 |
|  | HO2 | *C19* | 42 | 42 | 12 | 30 | 29 |
|  | M10 | *C1* | 42 | 41 | 7 | 34 | 32 |
|  | M10 | *C19* | 42 | 42 | 5 | 37 | 31 |
| High-density: | HO2 | *C1* | 42 | 41 | 17 | 24 | 21 |
|  | HO2 | *C19* | 42 | 42 | 18 | 24 | 24 |
|  | M10 | *C1* | 42 | 41 | 6 | 35 | 32 |
|  | M10 | *C19* | 42 | 34 | 4 | 30 | 27 |
| **Maternal effect of density signal manipulations** | | | | | |  |  |
| Low-density: | HO2 | *C1* | 42 | 38 | 4 | 34 | 32 |
|  | HO2 | *C19* | 42 | 41 | 9 | 32 | 31 |
|  | M10 | *C1* | 42 | 41 | 12 | 29 | 28 |
|  | M10 | *C19* | 42 | 41 | 15 | 26 | 26 |
| High-density: | HO2 | *C1* | 42 | 34 | 5 | 29 | 29 |
|  | HO2 | *C19* | 42 | 33 | 6 | 27 | 25 |
|  | M10 | *C1* | 42 | 40 | 7 | 33 | 33 |
|  | M10 | *C19* | 42 | 40 | 12 | 28 | 28 |
